# 

**Published:** 1911-01-28

**Authors:** 


### CONTENTS.

| | PAGE |
|---|---|
| **Medical Charges** | 519 |
| **The Reform of Medical Education in France** | 520 |
| **Annotations**—Sir Francis Galton—The Education and Examination of Pharmacists—A Nice Point | 521 |
| **Medical Opinion and Movement** | 522 |
| **Hospital Clinics**—Some Points in the Treatment of Scarlet Fever | 525 |
| **Special Articles**—The Reform of Medical Education in France | 527 |
| **Home and Continental Spas**—Bath | 529 |
| **Surgery**—The Operative Treatment of Fractures | 533 |
| **Practical Notes on Surgical Diagnosis and Treatment** | 534 |
| **Resident Medical Officers' Department**—The Treatment of Corneal Ulcers | 535 |
| **Tropical Medicine**—The Venom of Scorpions | 536 |
| **Editor's Letter-Box**—Aortic Compression—Medical Portraiture | 538 |
| **Hospital Architecture and Construction**—The Ward Floor | 541 |
| **Special Institutional Articles**—II.—Hospitals and the Law in 1910 | 543 |

